# Ultrasonic-Assisted Extraction of Phenolic Compounds, Flavonoids, and Antioxidants from Dill (*Anethum graveolens* L.)

**DOI:** 10.1155/2022/3848261

**Published:** 2022-12-02

**Authors:** Khakhanang Ratananikom, Kantapon Premprayoon

**Affiliations:** ^1^Department of Science and Mathematics, Faculty of Science and Health Technology, Kalasin University, Kalasin, Thailand; ^2^Department of Agricultural Machinery Engineering, Faculty of Engineering, Rajamangala University of Technology Isan, Khon Kaen Campus, Khon Kaen, Thailand

## Abstract

This study evaluated the effect of ultrasonic-assisted extraction (UAE) on the isolation of phenolic compounds, flavonoids, and antioxidants from dill. UAE improved the extraction yields of total phenolic compounds and total flavonoid content as well as increased the antioxidant activities of all dill extracts. The optimum UAE condition to obtain highest total phenolic compounds, total flavonoid content, and antioxidant activities was 50% ethanol for 30 min giving 135.88 ± 3.23 mg gallic acid equivalent/g extract and 229.53 ± 4.97 mg rutin equivalent/g extract, respectively. Lowest IC_50_ values against 2,2′-azino-bis (3-ethylbenzothiazoline-6-sulfonic acid) (ABTS) and 2,2-diphenyl-1-picrylhydrazyl (DPPH) radicals were 0.034 ± 0.00 mg/mL and 0.12 ± 0.00 mg/mL, respectively. Results indicated the capability of UAE in extracting biologically active compounds from dill as a prospective functional material.

## 1. Introduction

Various secondary metabolites including phenolic compounds, flavonoids, carotenoids, terpenoids, and alkaloids have been found in plants at high concentration. These bioactive compounds are essential to the plant's defense mechanism and act as antioxidants, antimicrobials, and nutrients that can be used to treat and prevent a variety of diseases for humans. Specifically, the most abundant bioactive constituents identified in edible plants are flavonoids and phenolic compounds, which serve as non-enzymatic antioxidants in cells to prevent damage from oxidative stress, making them abundant sources of metabolites that support human health [1, 2].

Examining extraction techniques is crucial for successfully obtaining these natural bioactive substances. Several methods for isolating natural bioactive compounds have been proposed. Conventional methods, such as Soxhlet extraction, reflux extraction, maceration, infusion, and percolation, are commonly used; however, these applications are time-consuming and involve high labor costs. Further, large amounts of extraction solvent are not environmentally friendly. Therefore, green alternative methods have been recently introduced to overcome these drawbacks; such techniques include ultrasonic-assisted extraction, microwave-assisted extraction, and supercritical fluid extraction. Ultrasonic-assisted extraction (UAE), among others, has enormous potential in the food and herbal industries because of its practicality and cost effectiveness. Additionally, it requires less time and energy, allows for low-temperature extraction, and maintains quality of the extract. UAE uses high-intensity sound waves to extract bioactive substances from their natural sources. Due to the acoustic cavitation generated by UAE, plant tissues are destroyed, and thus the contact area between solvent and plant materials is increased, enhancing higher extraction yield of bioactive compounds compared with conventional extraction methods [3–7].

This paper focuses on applying the UAE technique to extract phenolic compounds, flavonoids, and antioxidants from dill. Dill (*Anethum graveolens* L.) is an important herbal plant belonging to the Apiaceae family; it is used as a local spice in Central and Southwest Asia, Southeast Europe, and the Mediterranean regions, although it can be grown around the world [8]. Widely cultivated and consumed in Northeastern Thailand [9], dill (or “Phak Chi Lao” in Thai) has long been used in Asian conventional medicine and has been an essential element of customary Thai medication [9–11]. Previous studies have reported health benefits and pharmaceutical properties found in dill fruit, essential oil, and leaves, such as antioxidant activity, antimicrobial activity, diuretic property, carminative property, and appetite stimulant [9–13].

This paper makes a significant contribution by being the first to evaluate the use of the UAE technique to extract biologically active compounds from dill, one of the most ubiquitous plants in traditional Thai medicine and cuisine. Specifically, total phenolic compounds, total flavonoid content, and antioxidant activities were investigated to validate the optimum UAE condition for dill.

## 2. Materials and Methods

### 2.1. Materials

Dill leaves and stems were purchased from three local markets in Kalasin Province, Thailand. After cleaning with tap water, the edible parts were collected, dried by a freeze dryer, and then ground into powder.

#### 2.1.1. Ultrasonic-Assisted Extraction Procedure

Ultrasonic-assisted extraction was performed using an ultrasonic water bath (LabTech, Korea) at power and frequency of 350 W. and 50 Hz, respectively. Extraction variables of solvent concentration and time were investigated. Dried dill samples were extracted with ethanol (0, 50, 75, and 95% v/v), with sample: solvent ratio fixed at 1 : 10. Extraction times were set at 15 and 30 min. Liquid fractions were collected after centrifugation at 12,000 rpm for 10 min and concentrated by a rotary evaporator.

To compare UAE with conventional methods (CE), the maceration method was employed to set up the conventional method. It was conducted in shaking incubator at 30°C and 150 rpm for 30 min and using 50% ethanol as extraction solvent.

#### 2.1.2. Total Phenolic Compounds (TPCs)

The Folin–Ciocalteu method was used to determine TPC [14]. A 20 *µ*L aliquot of dill extract was mixed with 500 *µ*L of 10% Folin–Ciocalteu reagent and incubated at room temperature for 10 min. Then, 1 mL of 7.5% sodium carbonate was added, and the mixture was mixed and further incubated for 60 min at room temperature. Absorption at a wavelength of 765 nm was evaluated. TPC was expressed as mg gallic acid equivalent (GAE)/gram of extract by comparison with the gallic acid standard curve.

#### 2.1.3. Total Flavonoid Content (TFC)

Total flavonoid content was determined following the method of Wolfe et al. with some modifications [15]. A 200 *µ*L aliquot of dill extract was mixed with 150 *µ*L of 5% sodium nitrate and incubated at room temperature for 5 min. Then, 150 *µ*L of 10% aluminum chloride was added. After 6 min incubation at room temperature, 500 *µ*L sodium hydroxide was added to the mixture. Total volume was adjusted to 1.5 mL with 500 *µ*L of distilled water. Absorption at a wavelength of 510 nm was evaluated. TFC was expressed as mg rutin equivalent (RE)/gram of extract by comparison with the rutin standard curve.

#### 2.1.4. Antioxidant Activities

ABTS (2,2′-azino-bis (3-ethylbenzothiazoline-6-sulfonic acid) was used to determine the antioxidant activity of dill extracts following the method of Re et al. with some modifications [16]. To generate the ABTS cation radicals, 2.45 mM potassium persulfate and 7 mM ABTS in distilled water were mixed at a ratio of 1 : 1. The mixture was kept in darkness at room temperature for 16 hours to obtain the ABTS cation radicals. The ABTS cation radicals were then diluted with distilled water to obtain an absorbance of 0.8 ± 0.02 at 734 nm before use. Various concentrations of dill extracts and ABTS cation radicals were mixed and incubated in darkness at room temperature for 30 min. Absorption was measured at a wavelength of 734 nm. Antioxidant activities of the dill extracts were calculated using the following formula:

(1) $$\begin{array}{c} \text{ABTS scavenging activity }\left(\text{\%}\right)=\left[\frac{\left(\text{Abs}{}_{c}-\text{Abs}{}_{s}\right)}{\text{Abs}{}_{c}}\right]\times 100\text{,} \end{array}$$

where Abs_c_ and Abs_s_ are the absorbances of the control (without dill extract) and sample (with dill extract), respectively. Linear regression of antioxidant activity was used to determine the concentration of dill extract that could reduce the concentration of ABTS radicals by 50% (IC_50_).

2,2-Diphenyl-1-picrylhydrazyl (DPPH) was used to determine the antioxidant activity of dill extracts according to the method described by Brand-Williams et al. with some modifications [17]. An aliquot of 0.1 mM DPPH radicals in ethanol was mixed with various concentrations of dill extracts. The mixtures were kept in darkness for 30 min at room temperature. Absorption at a wavelength of 517 nm was measured. Antioxidant activities of the dill extracts were calculated using the following formula:

(2) $$\begin{array}{c} \text{DPPH scavenging activity }\left(\text{\%}\right)=\left[\frac{\left(\text{Abs}{}_{c}-\text{Abs}{}_{s}\right)}{\text{Abs}{}_{c}}\right]\times 100\text{,} \end{array}$$

where Abs_c_ and Abs_s_ are the absorbances of the control (without dill extract) and sample (with dill extract), respectively. Linear regression of antioxidant activity was used to determine the concentration of dill extract that could reduce the concentration of DPPH radicals by 50% (IC_50_).

#### 2.1.5. Statistical Analysis

Data were expressed as mean ± standard deviation. Data with normal distribution were analyzed using one-way analysis of variance (ANOVA), followed by least significant difference (LSD) with a significance level of *α* = 0.05.

## 3. Results

### 3.1. Total Phenolic Compounds and Total Flavonoid Content of Dill Extracts

Total phenolic compounds and total flavonoid content of dill extracts are summarized in Table 1. TPC and TFC of dill extracts differed according to the UAE condition. Yields of TPC and TFC increased when ethanol concentration increased to reach the highest at 50% ethanol as 73.94 ± 0.28 mg GAE/g extract and 119.69 ± 3.05 mg RE/g extract, respectively, when extraction time was fixed at 15 min. Reduction in both TPC and TFC yields was noticed when ethanol concentration increased from 50% to 95%. Extending the extraction time from 15 min to 30 min did not cause negative feedback on TPC and TFC yields. Significantly highest yields of TPC and TFC were 135.88 ± 3.23 mg GAE/g extract and 229.53 ± 4.97 mg RE/g extract, respectively, when using 50% ethanol as extraction solvent with 30 min of UAE.

### 3.2. Antioxidant Activity

**Figure 1:**
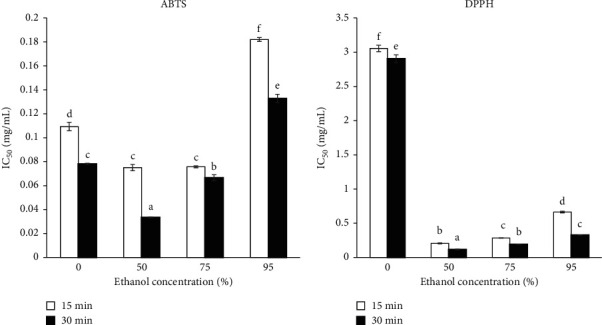
Antioxidant activity of dill extracts.

The antioxidant activities of dill extracts against ABTS and DPPH radicals were also investigated and presented as IC_50_ values in Figure 1. The IC_50_ value indicates the concentration of dill extract that could reduce the concentration of ABTS or DPPH radicals by 50%. The IC_50_ results agreed well with the TPC and TFC results. Significantly lowest IC_50_ values for both ABTS and DPPH radicals were found from dill extracts obtained using 50% ethanol for 30 min of UAE as 0.034 ± 0.00 mg/mL and 0.12 ± 0.00 mg/mL, respectively, while a longer period of UAE did not cause negative feedback on antioxidant activities.

### 3.3. Comparison of UAE and CE

To compare the efficiency of UAE with CE, samples obtained from CE (without ultrasonic treatment) were evaluated for their TPC, TFC, and the antioxidant activity. The results are presented in Table 2. Yield of TPC and TFC was significantly higher using UAE, compared to those of CE. TPC and TFC of UAE were 135.88 ± 3.23 mg GAE/g extract and 229.53 ± 4.97 mg RE/g extract, respectively, while CE gave TPC and TFC as 2.67 ± 0.27 mg GAE/g extract and 3.31 ± 0.21 mg RE/g extract, respectively. Also, the antioxidant activities against ABTS and DPPH radicals of UAE were significantly higher than those of CE. The IC_50_ value against ABTS and DPPH radicals of UAE was 0.034 ± 0.00 mg/mL and 0.12 ± 0.00 mg/mL, respectively, while the IC_50_ value against ABTS and DPPH radicals of CE was 4.43 ± 0.07 and 4.94 ± 0.03 mg/mL, respectively.

## 4. Discussion

The biologically active compounds, including flavonoids, phenolic compounds, saponins, cardiac glycosides, and terpenes, have various health benefits and can be natural antioxidants that can neutralize free radicals and reactive oxygen species (ROS). Free radicals and ROS can attack biomolecules and healthy cells to lose their structures and functions, causing oxidative damage of DNA, proteins, lipids, and other biomolecules which lead to a variety of diseases. Thus, receiving the adequacy of these biologically active compounds, derived from potential plants either by direct consuming or dietary supplement, is the most effective way to prevent the oxidative damage of various biomolecules [9–13, 18]. Therefore, finding a suitable and applicable extraction method to obtain these biologically active compounds is of interest.

According to our study, the edible plant portions of dill were a good source of phenolic compounds, flavonoids, and antioxidants. UAE greatly increased extraction efficiency and generated dill extracts with very highly active ingredients. For dill extracts, using 50% ethanol for 30 min was the optimum UAE condition. Yields of TPC and TFC, measuring as 135.88 ± 3.23 mg GAE/g extract and 229.53 ± 4.97 mg RE/g extract, respectively, were found to be approximately 50 and 69 times higher than those of CE. The scavenging ability against ABTS and DPPH radicals significantly increased while extracting under UAE conditions; the IC_50_ reduced to 0.034 ± 0.00 mg/mL and 0.120 ± 0.00 mg/mL, respectively, which were about 130 and 41 times lower than those of CE. Comparing TPC, TFC, and antioxidant activities from the dill extract with UAE treatment to those of other plant species, they were considerable. In the study by Saeed et al., TPC and TFC were evaluated from an extract of bristle fruit hedge parsley (*Torilis leptophylla*), which belongs to the same family as dill, the Apiaceae family. It was extracted by methanol via maceration technique for 2 days. The TPC and TFC of the methanol extract were 121.9 mg GAE/g extract and 59.6 mg RE/g extract, respectively, according to their research. In addition, it had IC_50_ values of 0.189 and 0.179 mg/mL for scavenging the ABTS and DPPH radicals, respectively [19].

The improvement in TPC, TFC, and antioxidant activities of dill extracts was attributed to the pervading nature of the ultrasonic waves that produced unstable cavitation bubbles which formed high-velocity jets upon collapsing [20]. This cavitation effect caused cellular disruption, with reduced material size that facilitated extraction solvent penetration. Physical properties of the extraction solvent such as concentration, polarity, viscosity, surface tension, and saturation vapor pressure have also been considered to take part in the cavitation effect, promoting higher UAE [5, 7]. Higher yields of TPC and TFC as well as improved antioxidant activities have been reported from several plants using UAE [6, 21–24].

Our results indicated that the UAE efficiency of dill extract was influenced by both solvent concentration and extraction time. In this study, ethanol and water were chosen as the extraction solvents due to safety and health considerations. Our findings were consistent with earlier studies, which indicated that employing a binary solvent system was preferable to a monosolvent system [25–27]. Using 50% ethanol provided the best extraction conditions for TPC, TFC, and antioxidants from dill. This result agreed with several studies that identified aqueous mixtures of ethanol at 50–70% as the suitable condition for the extraction of TPC and TFC because it could quickly infiltrate into plant cells more effectively than using pure water and pure extraction solvents [23, 28–30]. This evidence might be explained by the fact that both ethanol and water play distinct roles in extraction. Depending on the amount of ethanol present, ethanol forms a concentration gradient that promotes the diffusion of the solvent into the solute and, as a result, enhances mass transfer. Additionally, water helps the solute expand, while ethanol disrupts polyphenolic bonds in the solute [31]. Hence, the greater TPC and TFC output seen in this study was likely the result of a combination of these two occurrences.

Extension of UAE time to 30 min did not cause oxidation of TPC, TFC, and antioxidants. This result was in line with earlier research which found that TPC and TFC extraction efficiency was increased by longer UAE times, whereas an extension of the UAE treatment to 40–60 min could have negative effects by enabling the active compounds to oxidize and get destroyed [22, 26, 28]. The reason for increase of TPC and TFC via using 30 min UAE treatment could be attributed to the fact that UAE softens plant tissue, compromises the strength of cell walls, and increases the solubility of phenolic compounds, all of which would increase TPC and TFC dissociation in solvent.

To assess the free radical scavenging capacity of diverse substances, the ABTS and DPPH radical scavenging capacity assay is widely utilized. The ABTS and DPPH assays showed the increasing scavenging activity trends when the extraction time increased at all ethanol concentration. In this study, 50% ethanol and 30 min condition were considered the most effective way for dill extraction. This situation could be explained by the fact that the solubility of antioxidants depends on the chemical structures and the polarity of the solvent system. The estimation of ABTS and DPPH radical scavenging capacities is based on the capacity of existing compounds to scavenge ABTS and DPPH radicals. Our findings demonstrated the influence of solvent selection on the effectiveness of antioxidant extraction. It was possible that antioxidants, which are less hydrophilic, could not be extracted using pure water, or in other words, using 0% ethanol, while more hydrophilic antioxidants were more easily released when there is water present during the extraction. Therefore, using a mixture of ethanol and water would be more appropriate in isolating a wide range of antioxidants from dill.

However, applying UAE to extract active compounds from nature was not successful for all cases. In the study by Ramli et al. for instance, it was discovered that UAE dramatically increased the flavonoid content and scavenging activity for the peel of red dragon fruit while significantly decreasing the extraction yield. UAE, on the other hand, boosted extraction yield while lowering flavonoid concentration and scavenging activity for flesh of red dragon fruit [32]. This demonstrated that the physical characteristics of the sample had an impact on effect of the extraction method. As a result, validation and estimation of UAE optimum condition in terms of solvent concentration and UAE period for individual plants should be studied. Other UAE-related factors including frequency, power, duty cycle, temperature, and liquid solid ratio would be crucial to UAE efficiency and require careful management for the optimum extraction [7]. However, this work first emphasized the use of UAE to effectively extract biologically active compounds from dill, and 50% ethanol and 30 min condition were considered the optimized method for dill extraction.

## 5. Conclusions

Our investigations indicated that UAE assisted the extraction process of TPC, TFC, and antioxidants from dill. The optimum UAE condition for dill extraction was 50% ethanol and 30 min. Highest yields of TPC and TFC were 135.88 ± 3.23 mg GAE/g extract and 229.53 ± 4.97 mg RE/g extract, respectively, with lowest IC_50_ values against ABTS and DPPH radicals as 0.034 ± 0.00 mg/mL and 0.12 ± 0.00 mg/mL, respectively.

## Figures and Tables

**Table 1:** Total phenolic compounds and total flavonoid content of dill extracts.

Ethanol (%)	Total phenolic compounds (mg GAE/g extract)		Total flavonoid content (mg RE/g extract)	
	Time (min)		Time (min)	
	15	30	15	30
0	37.46 ± 0.83^g^	43.97 ± 1.22^e^	43.05 ± 0.20^g^	55.02 ± 1.30^e^
50	73.94 ± 0.28^c^	135.88 ± 3.23^a^	119.69 ± 3.05^c^	229.53 ± 4.97^a^
75	61.61 ± 1.69^d^	93.24 ± 1.71^b^	114.00 ± 2.55^d^	169.38 ± 1.67^b^
95	32.64 ± 0.40^h^	40.70 ± 0.14^f^	47.82 ± 0.64^f^	58.62 ± 1.67^e^

Remark: mean values with different letters within each group are significantly different (*P* < 0.01).

**Table 2:** Total phenolic compounds, total flavonoid content, and antioxidant activity of dill extracts from UAE and CE.

Method	Total phenolic compounds	Total flavonoid content	IC_50_ (mg/mL)	
	(mg GAE/g extract)	(mg RE/g extract)	ABTS	DPPH
UAE	135.88 ± 3.23^a^	229.53 ± 4.97^a^	0.034 ± 0.00^b^	0.12 ± 0.00^b^
CE	2.67 ± 0.27^b^	3.31 ± 0.21^b^	4.43 ± 0.07^a^	4.94 ± 0.03^a^

Remark: mean values with different letters within each group are significantly different (*P* < 0.01).

## Data Availability

The data used to support the findings of this study are included within the article, and further inquiries can be directed to the corresponding author.
